# Improving ethanol tolerance of ethyl carbamate hydrolase by diphasic high pressure molecular dynamic simulations

**DOI:** 10.1186/s13568-023-01538-7

**Published:** 2023-03-15

**Authors:** Qijia Zan, Mengfei Long, Nan Zheng, Zehua Zhang, Huimin Zhou, Xinjie Xu, Tolbert Osire, Xiaole Xia

**Affiliations:** 1 Key Laboratory of Industrial Biotechnology, Ministry of Education, School of Biotechnology, Jiangnan University, Wuxi, 214122 Jiangsu China; 2 Faculty of Biology, Shenzhen MSU-BIT University, Shenzhen, 518172 Guangdong China

**Keywords:** Ethyl carbamate hydrolase, Ethanol tolerance, Diphasic high pressure molecular dynamic simulations, Immobilization

## Abstract

Ethyl carbamate (EC) is mainly found in fermented foods and fermented alcoholic beverages, which could cause carcinogenic potential to humans. Reducing EC is one of the key research priorities to address security of fermented foods. Enzymatic degradation of EC with EC hydrolase in food is the most reliable and efficient method. However, poor tolerance to ethanol severely hinders application of EC hydrolase. In this study, the mutants of EC hydrolase were screened by diphasic high pressure molecular dynamic simulations (dHP-MD). The best variant with remarkable improvement in specific activity and was H68A/K70R/S325N, whose specific activity was approximately 3.42-fold higher than WT, and relative enzyme activity under 20% (v/v) was 5.02-fold higher than WT. Moreover, the triple mutant increased its stability by acquiring more hydration shell and forming extra hydrogen bonds. Furthermore, the ability of degrading EC of the immobilized triple mutant was both detected in mock wine and under certain reaction conditions. The stability of immobilized triple mutant and WT were both improved, and immobilized triple mutant degraded nearly twice as much EC as that of immobilized WT. Overall, dHP-MD was proved to effectively improve enzyme activity and ethanol tolerance for extent application at industrial scale.

**Supplementary Information:**

The online version contains supplementary material available at 10.1186/s13568-023-01538-7.

## Key points

1. Diphasic high pressure molecular dynamic simulations were applied for ration engineering of ethyl carbamate hydrolase with improved ethanol tolerance.
2. The specific activity of H68A/K70R/S325N was 3.42-fold higher than WT.
3. The tolerance of triple mutant H68A/K70R/S325N to 20%(v/v) ethanol was increased to 41.16%, which was 5.02-fold higher than WT.

**Supplementary Information:**

The online version contains supplementary material available at 10.1186/s13568-023-01538-7.

## Introduction

Ethyl carbamate (EC) is a toxic substance with a potential carcinogenic risk and genotoxicity to human beings (Forkert 2010). It is produced during the storage and transport of a wide range of fermented foods. EC is reported to be carcinogenic (Qin et al. 2021), which could be enhanced by ethanol in alcoholic beverages (Sakano et al. 2002), thus the content of EC in alcoholic beverages such as yellow wine, white wine, wine, sake and brandy has attracted increasing attention. The limit for EC in yellow wine is down to 100 μg/L, while the concentration of EC in yellow wine brewing ranges from 100 to 750 μg/L (Chen et al. 2017), which leads to great food safety and health concerns for long-term drinkers. The main methods used to reduce EC in food are process optimization (Weber and Sharypov 2009), metabolic engineering (Wu et al. 2016) and enzymatic methods (Cerreti et al. 2016; Zhao et al. 2013). The former two strategies aim at modifying the substrate and production pathway of EC, however, they may suffer from incomplete reduction, low efficiency and food flavor damage.

Enzymatic control mostly involves application of acid urease (Gun et al. 2015) and EC hydrolases (Dong et al. 2022) to reduce EC, which results in effective and mild conversion of urea and EC into ethanol, carbon dioxide and ammonia (Gowd V et al. 2018; Mohapatra 2018). Urease, permitted to be added to alcoholic beverages in many countries, is effective in reducing urea (precursor of EC) to control EC level, but it is negatively unable to degrade synthesized EC. EC hydrolase could directly and efficiently hydrolyze EC, which has been heterologous expressed and applied (Zhao et al. 1994), however, it is extremely unstable in acidic or high ethanol context (Akutsu-Shigeno et al. 2006; Jia et al. 2020), limiting its practical application.

Currently, the main strategies for the modification of urethane hydrolases consist of rational design (Korendovych 2018), directed evolution (Cheng et al. 2015) and semi-rational design (Fan et al. 2018). Rational design focuses on predicting mutatable regions and building accurate mutants libraries for experimental validation, based on the enzyme structure and catalytic mechanism (Lehmann and Wyss 2001; Marshall et al. 2003), meanwhile, directed evolution, also known as random mutagenesis, typically uses error-prone PCR or DNA recombination to gernerate a large pool of mutants followed by specific high-throughput screeningto identify efficient mutants (Wong et al. 2006). As a combination of rational design and directed evolution, semi-rational design prefers to locate suitable mutation sites as modification targets, hence constructing high quality mutant libraries (Lutz 2010). Liu et. al improved activity of a bifunctional urease by semi-rational design of targeted amino acid sites with a high impact on enzyme activity (Liu et al. 2018). However, the affinity and hydrolysis capacity for urea of the bifunctional urease was much higher than that of EC, indicating its ability to hydrolyse EC could be compromised in the presence of both urea and EC when applied to actual wine samples.

Kang et al. improved the catalytic activity of AmdA, an amidase derived from *Agrobacterium tumefaciens*, by rational and semi-rational modification of its catalytic activity towards EC (Kang et al. 2021). The mutant G195A was obtained by multiple sequence comparisons to mine the targeted mutation sites and increased the enzymatic activity 3.75-fold that of the wild type. This method is a more laborious and limited approach to screening for mutants. The multiple sequence comparison method of screening for mutant sites can only modify the protein in the direction of the evolution of this class of enzymes as a whole, but not for the defective regions of the protein itself. Saturation mutations targeting amino acids in the vincinty of the triple active center only improve the enzymatic activity of the protein and do not effectively improve other enzymatic properties of the enzyme. How to obtain excellent organic solvent tolerance of enzymes based on meticulous modification is still unresolve.

Enzymes are susceptible to deactivation affected through the overall structure destabilization at high temperatures, while increasing pressure within a certain range could offset the effects of high temperatures on enzymes. Although the mechanism of pressure effect on stability has not yet been elucidated, its effect on the interaction between protein functional groups and water seems to be opposite to that of temperature, such as solvent hydration, system entropy and internal interactions (Eisenmenger and Reyes-De-Corcuera 2009). In our previous study, the motion trajectory of T1 lipase was analyzed with high-pressure molecular dynamics simulations. The effect of protein cavity responding to pressure was calculated and the amino acid sites with high impact on protein stability were selected for virtual saturation mutation. Finally, the mutants L18M, A190Y, A190L and A186L was more robust of than WT at the optimal temperature (Zhu 2020). Therefore, high-pressure molecular dynamics simulations could be an effective strategy for the targeted stability engineering. However, organic solvent tolerance modification of EC hydrolase based on high pressure molecular dynamics has not been reported yet.

Here in this study, EC hydrolase from *L. fusiformis* SCO2was engineered through high pressure based organic solvent molecular dynamics simulations to screen out unstable regions. Then, virtual saturation mutations were proceeded by multi-scale free energy evaluation. The enzymatic properties of candidates were experimently verified for validation of organic solvent tolerance and applied in immobilized and mock wine experiments. Our strategy could be expected to serve as a valuable reference for enzyme evolution of organic solvent tolerance.

## Materials and methods

### Strains, plasmids, and culture conditions

The gene of EC hydrolase from *L. fusiformis* SCO2 (GenBank ID: KU353448) screened in the mouse intestine was synthesized by Sangon Biotech, and was constructed into the plasmid pET-20b for expression in *Escherichia coli* BL21 (DE3). Plasmids harboring mutants was constructed by Fast Mutagenesis kit V2 with wild type of EC hydrolase plasmid severed as template. All of the primers were listed in Additional file 1: Table S1. Luria-Bertani (LB) media and Terrific Broth (TB) media were used for culture and expression of EC hydrolase and its derivatives. The recombinant strains were inoculated into 20 mL LB medium containing 100 μg/ml ampicillin, and was inoculated at 37 °C overnight. Then, 3% inoculation of the culture was transferred into 200 mL TB medium, which was cultured at 37 °C. A final concentration of 0.1 mmol/L IPTG was fed when the OD_600_ of the culture reached at 0.4 for extra18h at 25 °C.

### Virtual screening of mutants based on high pressure coupled diphasic MD simulation

The structure of EC enzyme was analyzed and modelled by AlphaFold 2 through its original protein sequence (Laurents 2022). The enzyme structure was simulated in Gromacs 5.0.3 with Gromacs 54a7 force filed (Berendsen et al. 1995). The simulated environments were set at pressures of 1 bar, 500 bar, 1000 bar, with 10% (v/v), 50% (v/v), 100% (v/v) of ethanol solution, respectively. The number of ethanol molecules added to the simulated system was calculated based on the ethanol concentration. 683 ethanol molecules were added in the 10% (v/v) simulated system, while 1195 ethanol molecules were poured at 50% (v/v). Appropriate number of Na^+^ were added into the system for charge neutralization. Two stages of position-limited pre-equilibrium simulations, NVT equilibrium simulations and NPT equilibrium simulations, were completed at 313 K in 100 ps. Then RMSD and RMSF were calculated to analyze the changes in the protein conformation at different ethanol levels and pressure conditions. The EC trajectory were sampled at 1 ns intervals, which were used to calculate the molecular volumes of the samples. The compression coefficient (*β*_T_) was calculated by the statistical mechanical relationship between the compression coefficient *β*_T_ and the volume fluctuations:

$$\, < \,\delta {\text{V}}^{{2}} \, > \,\, = \,{\text{k}}_{{\text{B}}} {\text{TV}}\beta_{{\text{T}}}$$

Where k_B_ is the Boltzmann constant, T is the absolute temperature, V is the intrinsic volume of the system and *β*_T_ is the isothermal compression coefficient (Meersman et al. 2006). The secondary structure of the protein was obtained by the space-division method of Voronoi tessellation (Voloshin et al. 2011). The structures with large differences in *β*_T_ were selected as the key regions with an impact on protein stability, and the amino acids in these regions were subjected to virtual saturation mutations. These mutants were evaluated by multiscale free energy calculations with FoldX (Schymkowitz et al. 2005) and I-mutant2.0 (Capriotti et al. 2005), of which mutants that were expectable to improve stability were filtered for further experiments.

MD and analysis of the triple mutant was carried out in a similar way described above. Different numbers of water molecules as well as ethanol molecules around the active center and mutation sites between WT and mutant were counted by Pymol. The hydrophilic and hydrophobic accessible surface area of the protein were calculated by Gromacs 5.1. Changes in bond energy interactions among WT and mutants were calculated by ring sites (https://ring.biocomputingup.it/submit) (Torshin et al. 2002). VMD was used for visualization of the distinction of the number of water and ethanol molecules around the protein before and after mutation.

### Enzymatic activity assay

The enzyme activity of EC hydrolase was measured by the generation of NH_3_ in the reaction (Liu et al. 2017). One unit of the enzyme activity was defined as 1 μmol of ammonia produced by degradation of EC per minute by the enzyme solution under the most optimal reaction conditions.

1 ml of enzyme solution was added into 1 ml of 3% (w/v) of EC solution react under 37 °C for 15 min. Then 1 ml of 10% trichloroacetic acid was added into the system to terminate the reaction. 1 ml of solution I (15 g phenol and 0.625 g sodium nitroprusside to 250 ml) and 1 ml of solution II (13.125 g sodium hydroxide and 7.5 ml sodium hypochlorite to 250 ml) were added into the reaction mixture for 20 min at 37 °C. The absorbance of samples was detected at 625 nm for measurement of content of NH_3_.

The enzyme activity was calculated as:

$${\text{enzyme activity}}\, = \,\Delta OD_{625} \times \,n\, \times \,k/15$$

where ∆OD_625_ is the difference between the absorbance values of the sample and the blank control, n is the dilution multiple of the enzyme solution, k is the inverse of the slope of the standard curve and 15 is the reaction time.

### Methods of enzyme purification

The strains were collected by centrifugation after expression and were resuspended in phosphate buffer solution. After sonication for 30 min at 4 °C, the supernatant was collected by centrifugation. An affinity chromatography nickel column was employed for purification of target enzymes linked with His tag. For purification, the unbound impurity protein was firstly eluted with 100 mM concentration of imidazole and the target protein is eluted at an imidazole concentration of approximately 300 mM. The collected eluted protein was concentrated and desalted through an ultrafiltration tube, which was used for enzymatic property determination with appropriate dilution.

### Determination of enzymatic properties

Protein concentrations were determined by a modified Bradford protein assay kit. Kinetic parameters of the WT EC hydrolyze and variants were determined with the EC concentration ranged from 0 to 300 mM. The relationship between enzymatic activities and substrate concentrations was plotted through Origin in order to fitting the Michaelis–Menten equation, from which *V*_max_ and *K*_m_ were obtained. *K*_cat_ was then calculated based on the equation: *K*_cat_ = *V*_max_/(E), where (E) represented the molar concentration. For the determination of optimum temperature, the enzyme activity was measured at different temperature conditions (30–60 °C). For validation of the stability, the enzyme was holden under different temperature (30–60 °C) for 30 min, and was measured the activity. The impact of ethanol on protein activity was determined through measurement of the enzymatic activity under different ethanol concentration. The stability of the protein in ethanol was measured under different concentrations of ethanol for 1 h. The half unfolding temperature (*T*_m_) of the wild type EC hydrolase and the mutant (chloride ions free) was analyzed by a MOS-450 circular dichroism spectrometer (Biologic, Franc), during which the temperature ranged from 20 to 80 °C.

### Simulated wine samples to determine the combined properties of the enzymes

The ability of the mutant and wild-type proteins to hydrolyze EC was verified in simulating a wine sample, which consisted of 500 ug/L of EC, 15% (v/v) ethanol at pH 4.5. A final concentration of approximately 1.5 mg/ml of protein was added to the mock wine sample yellow wine and the reaction was carried out at 30 °C for 12 h. The reaction solution was sampled after 1 h, 3 h, 7 h and 12 h for detection of residual EC by GC-MS (Thermo Fisher, America). 1 uL of diluted samples were injected into a J&W DB-WAX quartz capillary column (30 m × 0.25 mm, 0.25 um) with 1 mL·min-1 of gas (He) flow rate. The initial temperature of the heating procedure was set at 40 °C for 0.75 min, then gradually rose to be 60 °C in a speed of 10 °C·min-1. Afterwards, the temperature went up to 150 °C in a speed of 3 °C·min-1, and quickly soared to 220 °C for 4.25 min. Mass spectrum conditions were as follows: Electron Ionization Mode, energy: 70 eV, transmission line temperature 180 °C; ion source temperature 200 °C; selective ion scanning (SIM), qualitative ion m/z62, m/z74, m/z89, quantitative ion m/z62; the activation voltage was 1.5 V (Zhang et al. 2014). The amount of EC hydrolase consumed was calculated as the hydrolysis capacity of EC hydrolase by measuring the residual EC amount in the sample.

### Immobilization improves enzymatic properties

Chitosan was dissolved in 1.5% (v/v) acetic acid solution, stirred at high speed to form a 3% chitosan solution, and then ultrasonic oscillated overnight to remove bubbles. The chitosan solution was absorbed with a 10 mL syringe and added drop by drop to a solution containing 1 M KOH and 25% (v/v) ethanol, stirring at low speed. After the liquid drops were dropped, a small ball with a diameter of about 2 mm was formed. After the ball formed, left it for 2 h. Then, the small ball was taken out and washed in water thoroughly until the washing liquid is neutral. The chitosan pellets were placed in 1% glutaraldehyde solution to activate the surface groups at 37 °C for 3 h. After activation, the pellets were washed and placed in 4 °C phosphate buffer which contained wild-type EC hydrolase and H68A/K70R/S325N respectively, and cross-linked for 4 h for enzyme immobilization. At the end of crosslinking, the unbound enzyme was washed away with buffer solution. The immobilized pellets were stored in phosphate buffer at 4 °C. The optimal temperature, ethanol tolerance and ethanol stability of the immobilized protein were determined by methods similar to those used for the determination of free protein, except that the enzyme solution was replaced by three immobilized chitosan pellets. The repeated detection method for the determination of immobilized protein was to take three immobilized pellets and add EC solution for reaction. After the reaction, the filtrate was collected and the hydrolysis efficiency of the enzyme to EC was detected. After the ball was washed, the substrate solution was added again and the experiment was repeated for five times.

## Results

### EC hydrolase mutants screening based on dHP-MD

**Fig. 1:**
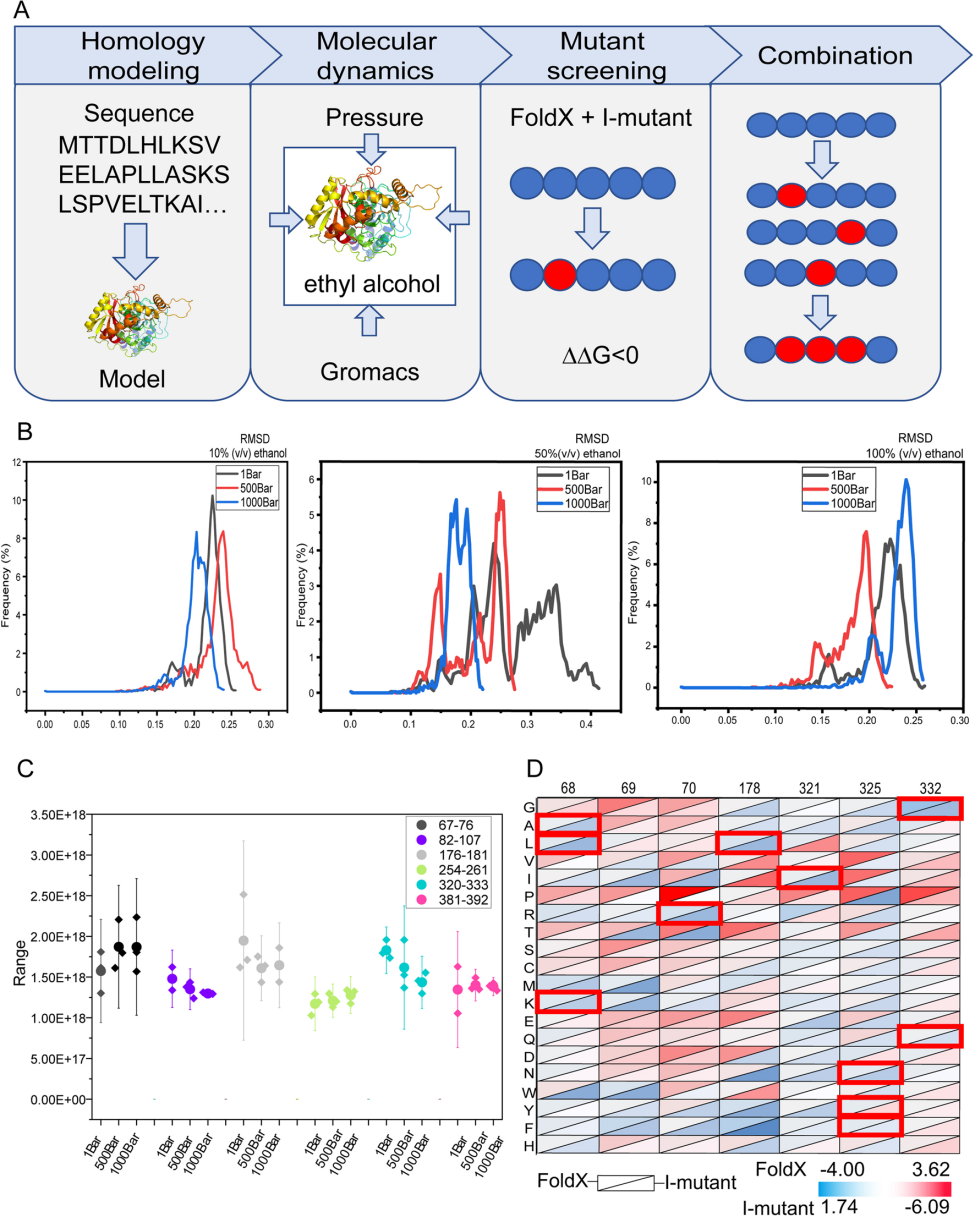
Mutant screening process. **A** Experimental procedure for screening mutants by modelling and simulating protein sequences and validating them in experiments. **B** RMSD of proteins at different ethanol concentrations and different pressures (10%, 50%, 100% ethanol concentrations, 1, 500, 1000 bar). **C** Compression coefficients of individual protein structures at different ethanol concentrations and different pressures. Each group represented a different loop structure, each line in the group represented a different pressure in the simulated environment, and each point on the line represented *β*_T_ at different ethanol concentrations. **D** Heat map of free energy changes at mutant sites obtained from multi-scale free energy calculations, the red box in the figure is the final selected results of the research

In this study, we combined the enzyme modification strategy based on isothermal compression micro-perturbation developed in our previous research (Zheng et al. 2022) with diphasic molecular dynamic simulation, named diphasic high pressure molecular dynamic simulations (dHP-MD), aimed at targeting enzyme mutants with improved activity and stability, especially organic solution resistance. A total of 1350 ns MD was performed (5 parallels × 3 pressures × 3 concentrations × 30 ns) under gradient pressures (1, 500, 1000 bar) and ethanol concentrations (10%, 50%, 100% v/v) was performed for exploration of the influence of ethanol molecules in structure of EC hydrolase and selection of ethanol-sensitive sites for further experiments (Fig. 1A). The distribution of root mean square deviations (RMSD) indicated the changes in flexibility of the protein during the simulation (Fig. 1B), which showed the greatest changes revealed under the condition of 50% (v/v) ethanol. In contrast, the changes in protein structure reduced at 100% (v/v) ethanol, probably due to the invasion of the protein structure by ethanol. The results indicated that appropriate ethanol concentration and pressure could expose flexible regions of the protein, thus making it easy to identify and screen the unstable regions. The root mean square fluctuation (RMSF) of protein structure under these conditions was also analyzed (Additional file 1: Fig. S1–S3), and the overall trend was similar to RMSD. The secondary structure α helix, β sheet and loop, the most basic units of enzyme spatial structures, was selected to classify the structure of EC hydrolase. Among the three secondary structures α helix, β sheet and loop, the conformational changes of loop were more intense than those of α helix and β sheet. Thus, we choose to screen out the region with large fluctuations from loop region for calculation, where the constant temperature compression coefficient *β*_T_ was introduced to evaluate the conformation response of the certain loops to ethanol. Six appropriate loop structures were selected for the calculation of *β*_T_, namely amino acids loop_67-76_, loop_82-107_, loop_176-181_, loop_254-261_, loop_320-333_ and loop_381-392_. Moreover, the regions loop_67-76_, loop_176-181_ and loop_320-333_ of EC hydrolase showed the highest *β*_T_ fluctuations that were regarded as “sensitive fluctuation” regions for further analysis (Fig. 1C). The amino acid residues in these three structures were subjected to virtual saturation mutation, and FoldX and I-mutant 2.0 were introduced to evaluate the changes in their stability after mutation. According to the thermodynamic hypothesis, the initial conformation of enzyme is the conformation with the lowest free energy (Zheng et al. 2020). The mutants of ΔΔG (ΔG mutant-ΔG WT)  < 0 obtained by FoldX based on the empirical effective energy function are the positive mutants with increased stability. In order to prevent more negative and neutral structures from being obtained by a single prediction program (Wijma et al. 2014), another prediction program, I-mutant 2.0, was also used to calculate the change of free energy, in which ΔΔG > 0 represents the stability of the mutant is increased. The final calculation results are shown in Fig. 1D, where the upper left corner of each cell represents the result of FoldX, the lower right corner represents the result of I-mutant 2.0. Finally, the mutants that showed increased stability in both predictions were A321I, S325F, S325Y, S325N, Q332E, Q332G, H68A, H68L, H68M, H68K, H68Y, K70R and A178L, and were used for further experimental validation.

### Characterization of the single-point mutations

**Fig. 2:**
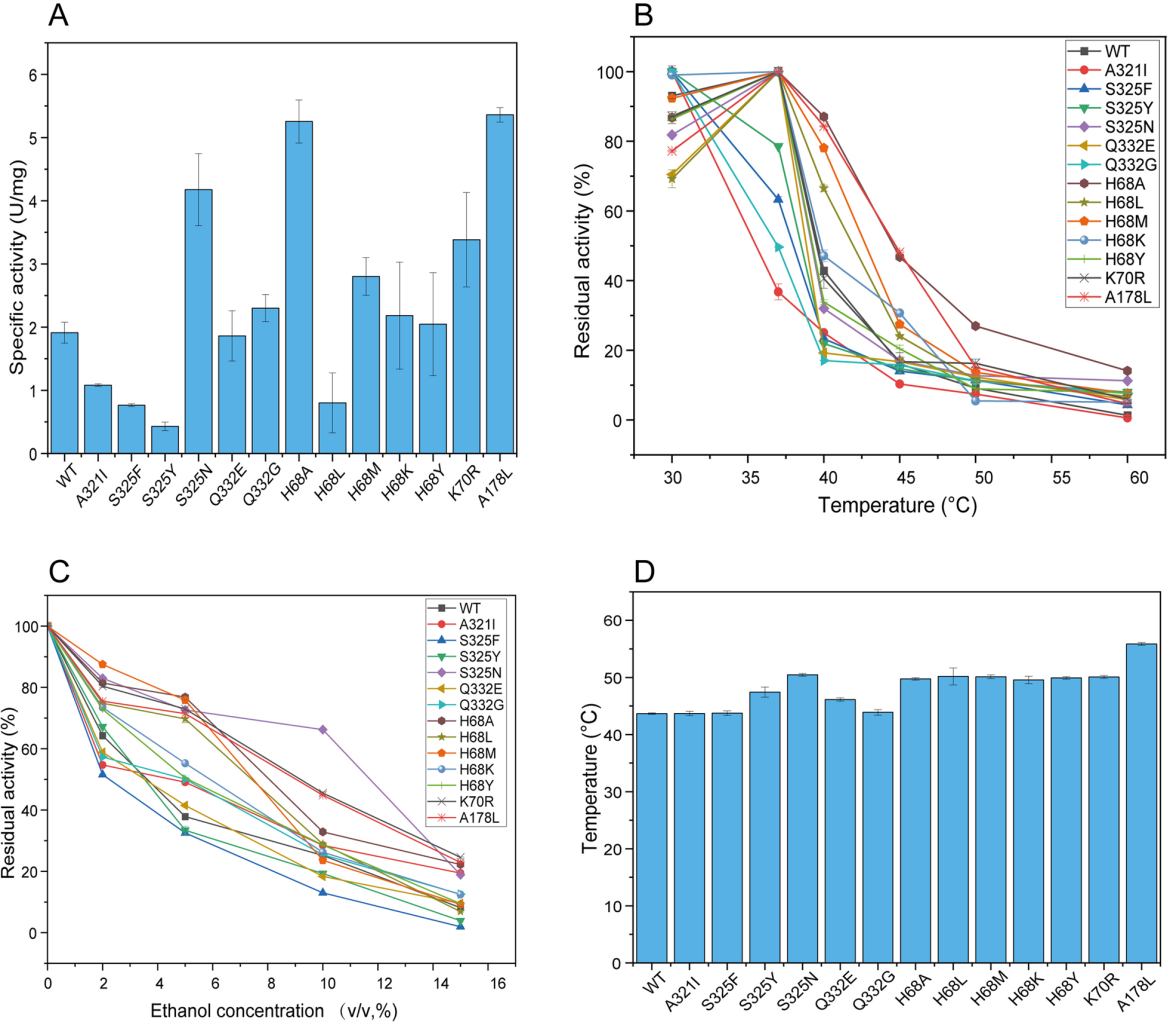
Enzymatic properties of single mutant proteins. **A** Specific enzymatic activity of single mutant proteins. **B** Optimal temperature of single mutant proteins. **C** Ethanol tolerance of single mutant proteins. **D** Variation in *T*_m_ values of single mutant proteins

All mutants were purified and assayed for specific enzyme activity (Fig. 2A). More than half of the mutants showed higher specific enzyme activity than WT. S325N, H68A, K70R and A178L showed significant increases, whose specific enzyme activity were 4.18 U/mg, 5.26 U/mg, 3.38 U/mg and 5.36 U/mg, respectively, compared to 1.89 U/mg in WT, which was an increase of about 2.21-fold, 2.78-fold, 1.79-fold and 2.84-fold. The specific enzyme activity of A321I, S325F, S325Y, H68L decreased compared to WT. Such as the specific enzyme activity of S325Y was only 0.43 U/mg, which was 0.23-fold than that of WT.

The optimum temperatures of most mutants were at 30 °C, which was lower than that of WT (optimum temperature at 37 °C) (Fig. 2B). But as the temperature rose above 40 °C, most mutants showed a slow trend in decreasing of enzyme activity and a higher relative activity than WT. The tolerance to temperature were improved in H68A and A178L. The relative enzyme activity of H68A at 50 °C and that of A178L at 45 °C were 26.90%, 48.31%, which were both higher than WT. In contrast, Q332E had the greatest decrease which was only 19.24% relative enzyme activity remained after reaction at 40 °C.

Most of the single point mutants showed an enhanced ethanol tolerance compared to WT (Fig. 2C). Among them, S325N, K70R and A178L showed an obvious improvement in relative enzyme activity, which were 2.62-fold, 1.80-fold and 1.78-fold higher than that of WT at 10% (v/v), respectively. Both mutant S325F and S325Y were less tolerant to ethanol than WT, with only 1.93% and 3.87% relative enzyme activities remaining after the reaction at 15%(v/v) ethanol, which was lower than WT (8.12%). The relative enzyme activities of H68L were higher than those of WT under 5% (v/v) and 10% (v/v) ethanol, but less than that of WT under 15% (v/v) ethanol.

A178L had improved the most, which elevated from 43.7 °C in WT to 55.9 °C (Fig. 2D). Furthermore, another mutant S325N also had a higher *T*_m_ value of 50.5 °C. The values of *K*_m_ and *K*_cat_/*K*_m_ for WT were 37.67 mM and 491.42 s^−1^·M^−1^ (Table 1), respectively, while the values of *K*_m_ and *K*_cat_/*K*_m_ for H68A and K70R were 31.38, 33.38 mM and 1589.33, 944.16 s^−1^·M^−1^, respectively, indicating that the affinity and catalytic efficiency of these two single point mutated proteins were improved. The kinetic constants of the rest of the mutants are listed in Additional file 1: Table S2.

**Table 1:** Enzymatic kinetic parameters of the three mutant proteins

	*K*_m_ (mM)	*V*_max_ (μmol·min^−1^.mg^−1^)	*K*_cat_ (s^−1^)	*K*_cat_ /*K*_m_ (s^−1^·M^−1^)
WT	37.67 ± 3.55	2.22 ± 0.07	18,509.92 ± 4341.41	491.42
S325N	45.52 ± 9.41	1.96 ± 0.17	16,366.99 ± 977.41	359.56
H68A	31.38 ± 5.61	5.98 ± 0.41	49,874.79 ± 2292.87	1589.33
K70R	33.38 ± 5.67	3.78 ± 1.01	31,517.63 ± 1408.41	944.16
A178L	96.78 ± 6.09	3.97 ± 1.18	33,146.88 ± 7908.62	342.48
H68A/S325N	33.27 ± 2.31	3.77 ± 0.97	26,180.56 ± 6736.11	786.91
K70R/S325N	40.82 ± 3.56	4.33 ± 0.54	17,182.54 ± 2142.86	420.93
H68A/K70R/S325N	28.2 ± 4.41	2.54 ± 0.029	15,484.74 ± 175.08	549.1

### Enzymatic properties of combinatorial variants

H68A, K70R, A178L and S325N were selected as the candidates applied in subsequent combinatorial mutation validation as well as the mutant Q328C which improved ethanol tolerance and thermal stability previously reported (Liu et al. 2016). Generally, 10 double mutants and 10 triple mutants were constructed to measure enzymatic performance.

**Fig. 3:**
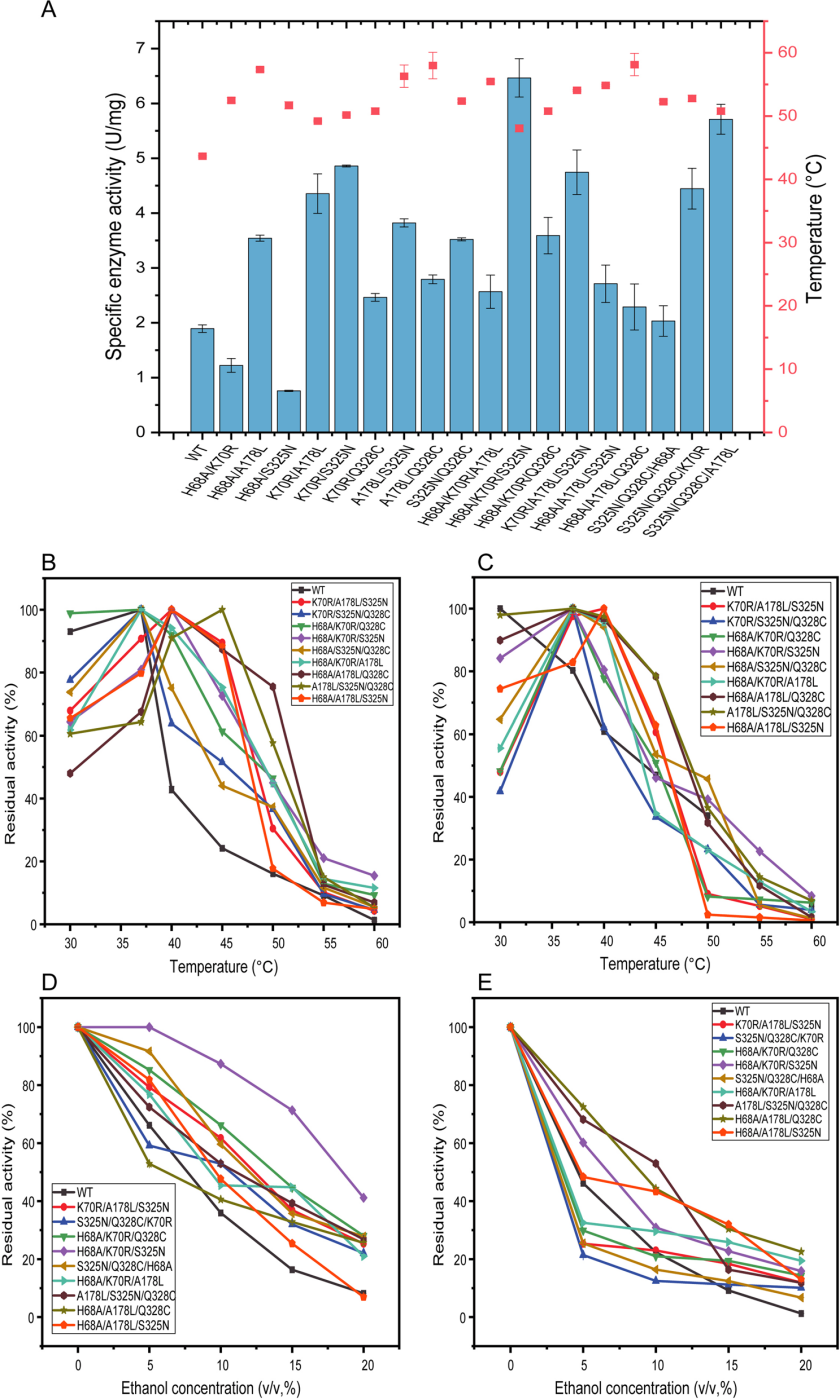
Diagram of the enzymatic properties of the three mutant proteins. (**A**) Specific enzymatic activity and *T*_m_ values of double and triple mutant proteins. **B** Optimal temperature of the triple mutant proteins. **C** Temperature stability of the triple mutant proteins. **D** Ethanol tolerance of the triple mutant proteins. **E** Ethanol stability of the triple mutant proteins

The specific enzyme activity of double mutants and triple mutants were general higher than that of WT (Fig. 3A), H68A/Q328C, K70R/A178L/Q328C had no enzyme activity. The results of some mutants were slightly less than that of WT, but the thermal stability and ethanol tolerance of mutants were increased greatly. In double mutants, the specific enzyme activity of K70R/A178L, K70R/S325N had a definite improved, which was 4.35 U/mg, 4.86 U/mg and was 2.30-fold, 2.57-fold than WT. Among triple mutants, a significant increase was shown in H68A/K70R/S325N, S325N/Q328C/A178L and H70A/A178L/S325N which was 6.46 U/mg, 5.71 U/mg, 4.74 U/mg, an increase of approximately 3.42-fold, 3.02-dold, 2.51-fold. The *T*_m_ value of H68A/K70R/S325N was also higher than that of double mutants (Fig. 3A), which was increased from 43.7 °C to 54.8 °C.

Most of the double mutants had a higher optimal temperature than WT (Additional file 1: Fig. S4). The optimal temperature of K70R/Q328C, A178L/S325N and A178L/Q328C was increased from 37 °C to 45 °C, and was the highest temperature among these variants. On the other side, when the temperature was 60 °C, H68A/K70R had a higher relative enzyme activity than other mutants, which was 12.03% and was 9.11-fold than WT.

H68A/K70R, K70R/A178L were sensitive to ethanol, they performed lower residual activity when ethanol existed than WT (Additional file 1: Fig. S5). On the contrast, H68A/S325N, K70R/S325N and A178L/S325N were the top three with the highest residual activity under 20% (v/v) ethanol, which were 32.42%, 27.73%, 25.74% and was 3.95-fold, 3.38-fold, 3.14-fold than that of WT. H68A/S325N showed higher affinity for the substrate than WT, meanwhile K70R/S325N showed higher catalytic efficiency than WT (Table 1).

Furthermore, triple mutants were constructed and analyzed to obtain mutants with higher stability and greater ethanol tolerance. In order to analyze the enzymatic properties more comprehensively, the determination of thermal stability and ethanol stability was added to the experiment (Fig. 3B–E).

Similar trends were shown in the optimum temperature and thermal stability with enzyme activity mostly decreasing above 40 °C. A slow decline was shown in the enzyme activity of A178L/S325N/Q328C with temperature increasing, both in optimum temperature and thermal stability assay. High ethanol tolerance and stability was shown in mutant H68A/K70R/S325N, whose relative enzyme activity was 41.16% after 15 min of reaction in 20% (v/v) ethanol, 5.02-fold higher than WT relative enzyme activity of 8.20%. Its relative enzyme activity was still 15.87%, in 20% (v/v) ethanol for 1 h.

The affinity for the substrate and catalytic efficiency of H68A/K70R/S325N was increased (Table 1). The overall performance of triple mutant H68A/K70R/S325N has improved compared to WT, which proved the success of mutant screening. A subsequent investigation, structural and molecular dynamics simulation analysis, was carried out to investigate why the ethanol tolerance and stability of H68A/K70R/S325N was improved.

### Structural analysis and molecular dynamics simulation of EC hydrolase variants

**Fig. 4:**
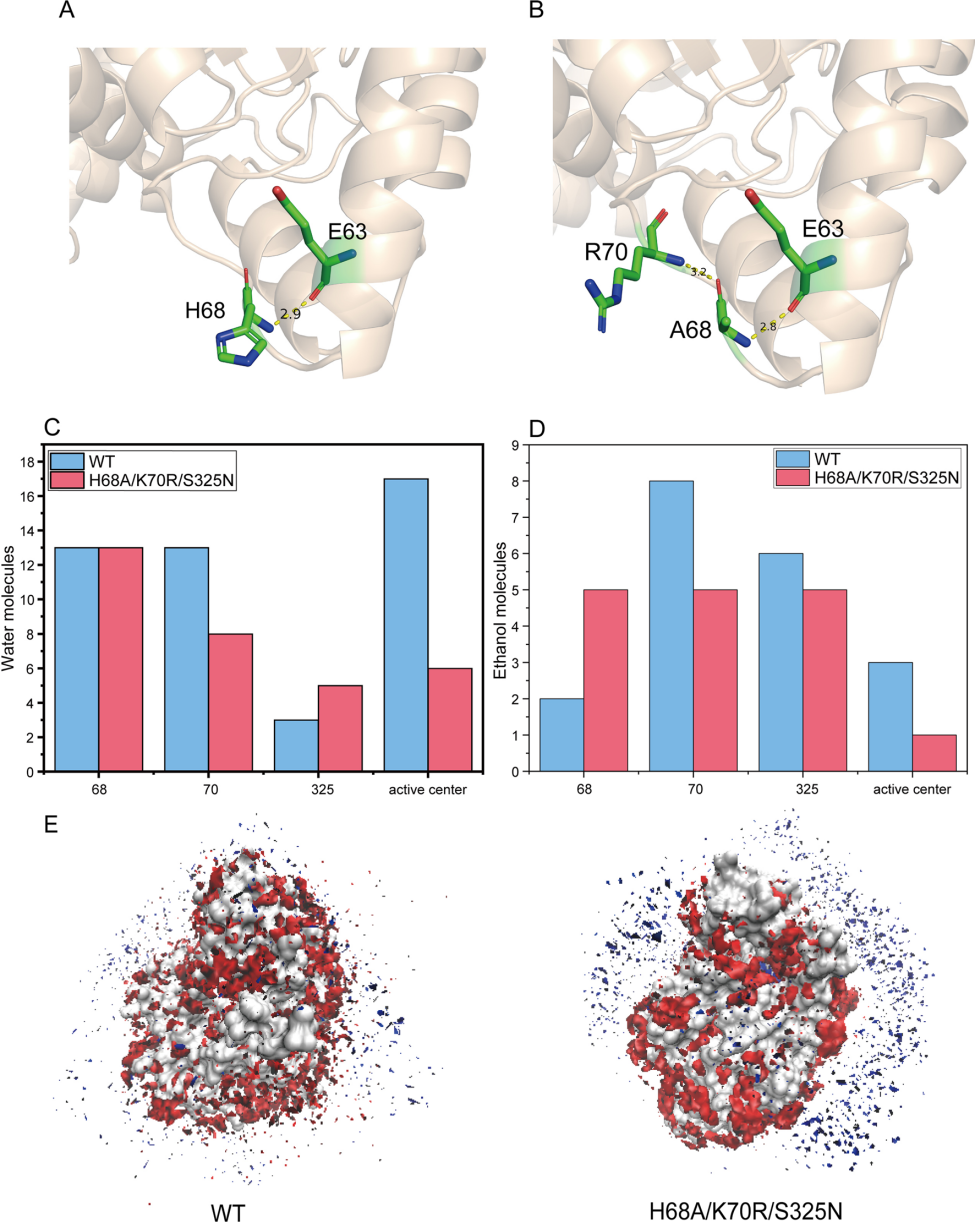
Structural and simulation analysis of the triple mutant protein in the ethanol system. **A** Hydrogen bonds around WT mutation residue **B** Hydrogen bonds around mutation residue of the triple mutant **C** Water molecules near the mutation sites and active center **D** Ethanol molecules near the mutation sites and active center **E** Water molecules and ethanol molecules around the wild-type and triple mutant proteins

In order to investigate the mechanism for improved ethanol tolerance of H68A/K70R/S325N, we focused on the internal molecular interaction changes between WT and the triple variant. The mutant not only retained the previous hydrogen bond between Ala68 and Glu63, but formed a new hydrogen bond between Ala68 and Arg70 (Fig. 4A, B). We also analyzed the mutant site Asn325 in the result of RMSF (Additional file 1: Fig. S6), in this site the mutant showed a less volatility than WT, which meant Asn325 had a greater stability than Ser325.

Furthermore, 50% (v/v) of ethanol-water diphasic of MD simulations were proceeded to uncover the fluctuation of hydration shell between EC hydrolase variants. We compared the number of ethanol molecules and water molecules near the mutation sites and active center of the triple mutant and WT to investigate the invasion of ethanol (Fig. 4C, D). The number of water molecules near Asn325 and the number of ethanol molecules near Ala68 increased in the triple mutant, while the number these molecules in the mutant at the other mutant sites and active center were less than that of WT. The invasion effect on enzyme activity by ethanol was weakened with reduced number of ethanol and water molecules at active center in triple mutant, which could protect enzyme activity in ethanol system. In addition, hydrophilic and hydrophobic accessible surface areas were also calculated (Additional file 1: Fig. S7), both of them in mutant were decreased within 30 ns under 50% (v/v) ethanol compared to WT. Results above suggested that the mutant enzyme formed a tighter contracted structure overall, thus the hydrated layer of the protein itself was protected and the impact of ethanol intrusion was reduced. These changes made overall structure of the protein more stable and less susceptible to changes in ethanol concentration and temperature, resulting in a higher enzyme activity than WT high ethanol concentrations. The amount of water and ethanol surrounding WT and mutant proteins was plotted by VMD (Fig. 4E). It can be seen that the number of ethanol molecules surrounding the mutant protein was reduced, indicating that the mutant protein had an increased affinity for water, thus protecting the enzyme from the influence of ethanol. The ethanol-sensitive sites in EC hydrolase were selected through dHP-MD, which were modified for mutants with ethanol resistance. The results showed that the triple mutant protected the hydration layer of the protein and enhanced the ethanol resistance. Interestingly, the balance between the activity and the stability of ethanol could be maintained, with improved activity as well as stability.

### Application of immobilized triple mutant

**Fig. 5:**
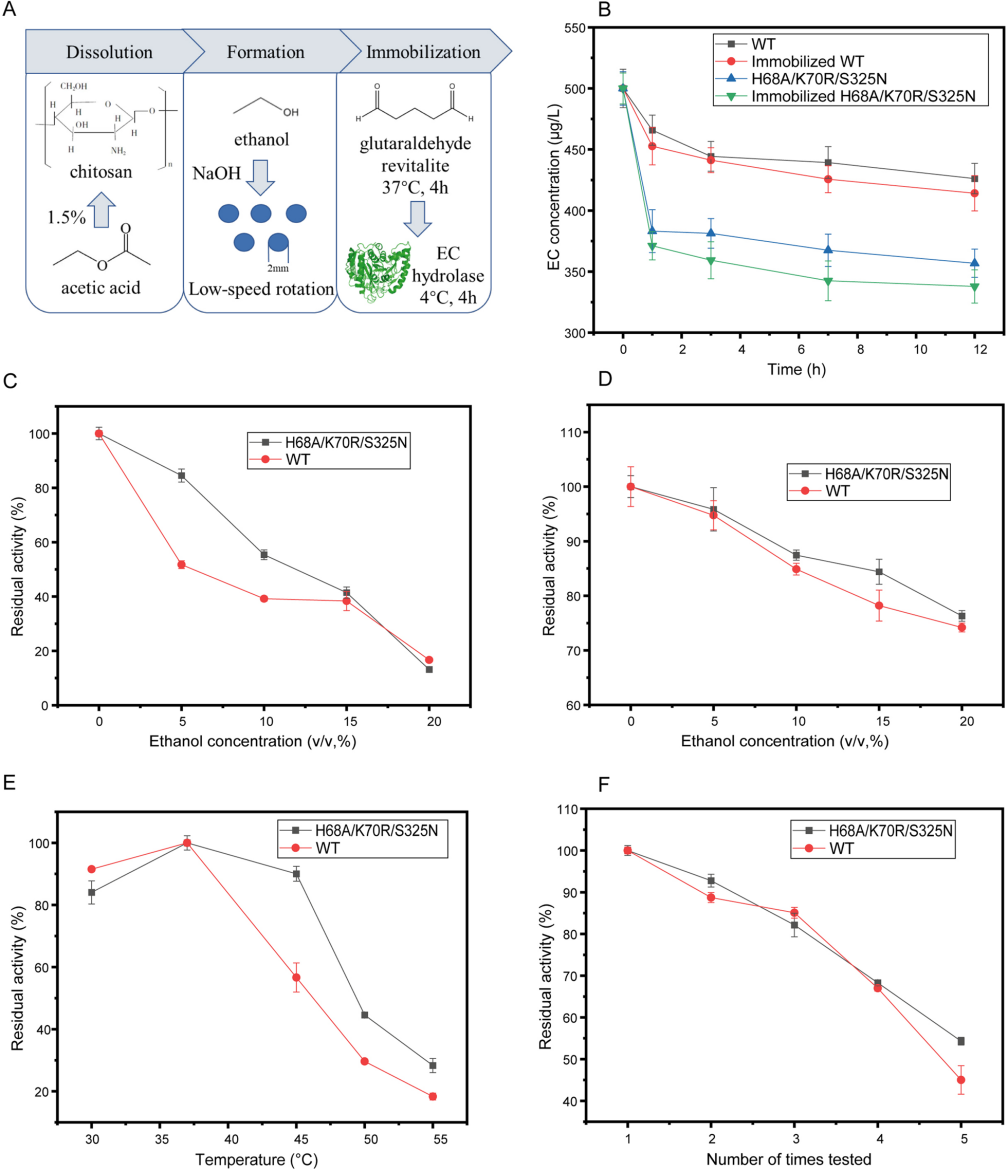
Determination and application of enzymatic properties after immobilization of EC hydrolase. **A** Enzyme immobilization **B** Application of WT and mutants in simulated wine samples **C** Ethanol stability of immobilized enzyme **D** Ethanol tolerance of immobilized enzymes **E** Optimum temperature for immobilized enzyme **F** Reuse of immobilized enzyme

Furthermore, immobilized EC hydrolase with chitosan was used for improvement of stability (Fig. 5A). Firstly, the degradation of EC by free and immobilized pellets was verified in simulated wine samples (500 μg/L EC, 15% ethanol, pH 4.5) (Fig. 5B). After the final concentration of 1.5 mg/ml free enzyme was added to the sample and degraded EC at 30 °C for 12 h, 14.81% EC was degraded by WT and 28.62% by free H68A/K70R/S325N. Alternatively, the immobilized WT enzyme degraded 18.01% EC in the simulated wine sample, and the immobilized mutants degraded 32.52% EC under the same conditions for 12 h. The results above showed that the triple mutant had better degradation ability of EC than WT under simulated wine conditions, while the immobilized enzyme performed better than free enzyme.

Meanwhile, other enzymatic properties of the immobilized EC hydrolase were determined. The activity of immobilized EC hydrolase decreased significantly after being kept in a certain amount of ethanol (5–20% v/v) for 1 h, (Fig. 5C), and the enzyme activity of H68A/K70R/S325N decreased slower than that of immobilized WT with the increase of ethanol concentration. However, the residual activity of the immobilized mutant was significantly increased in direct reaction in various concentrations of ethanol (Fig. 5D). The relatively residual activity of the immobilized mutant was 76.31% at 20% (v/v) ethanol, about 1.85-fold of that of the free triple mutant. The optimal temperature of the immobilized enzyme remained unchanged (Fig. 5E), but the enzyme activity of the mutant was increased to 44.61% at 50 °C, and the relative enzyme activity of immobilized WT was also increased to 29.63% at 50 °C. Meanwhile, the recovery rate for recycle was also determined (Fig. 5F). The residual enzyme activity of immobilized WT in the fifth experiment was 45.02%, while that of H68A/K70R/S325N was 54.31%. The above results showed that the immobilization successfully improved the stability and ethanol tolerance of the protein, and provided potential for practical application of EC degradation.

## Discussion

With the development and application of industrial biocatalysts technology, enzymes with organic solvent resistance have been pronouncing their great application potentials. However, the conformational change, the destruction of hydrophobic core and the reduction of catalytic rate of enzyme in organic solvents would easily lead to irreversible degeneration of enzyme (Bellissent-Funel et al. 2016). In general, organic solvents produced more opportunities for certain substrates and products with poor water solubility to react with enzymes for biocatalysts and/or food safety (Banik et al. 2016; Klibanov 2001). However, most of the enzymes showed poor stability and activity in organic solvent (Wang et al. 2016). Some studies had already discussed about probable reasons that organic solvents influenced the structure and function of enzymes, such as the organic solvent invasion caused the conformational change inside the enzyme (Mohtashami et al. 2019), water molecules stripped off (originally bond to the enzyme) by organic solvents (Serdakowski and Dordick 2008), changes in the solubility of the substrates (Klibanov 1997). Kamal et al. proved that methanol and isopropyl alcohol could make *Bacillus subtilis* lipase A unfold easier, and increase its instability (Kamal et al. 2013).

Therefore, how to improve the enzyme resistance to organic solvents has become one of research hotspots. Currently, there are three sorts of strategies reported to improve the organic solvent tolerance of enzymes: (i) Obtaining enzymes tolerant to organic solvent based on screening of those strains with organic solvent tolerance (Dyrda et al. 2019). However, the adaptive evolution for organic solvent tolerance is often changed by cell-resistant regulating element responding organic solvents with unsatisfactory mining of enzyme with organic solvent resistance. (ii) The immobilization technique of enzymes is used to improve the tolerance of enzymes in organic solvents. The enzymes act through covalent or non-covalent binding with enhanced structural rigidity in organic solvents by adsorbing themselves on vectors such as silicone gel, resistant hydroxylated polyketones, magnetic multi-pore metal materials (Yushkova et al. 2019). The tolerance of solvents such as dimethyl sulfoxide was significantly improved by the formation of amide bonds between urease and amino functional magnetic nanoparticles (Jangi et al. 2020). However, immobilized enzymes could cause partial inactivation of enzymes, change of enzyme kinetics, and requirement of enzyme separation and purification, which would increase its cost of production and application. (iii) Improving organic solvent tolerance of enzymes based on structural engineering. Enzyme engineering mainly transforms the structure of enzymes by changing amino acid residues, and could precisely modify the properties that need to be improved or the shortcomings of enzymes, taking the advantages of simple operation, low cost and high efficiency.

As for strategies for enhancing organic solvent tolerance of enzymes, rational design, semi-rational design and directed evolution had been employed for enzyme engineering. In previous research, Liu et al. (Liu et al. 2018) obtained the urease with increased affinity for EC by semi-rational design, which determined key “hot” regions that had great influence on enzyme activity to screen out mutants with improved enzymatic properties. Alternatively, Kang (Kang et al. 2021) screened EC hydrolase mutants with increased enzyme activity by multisequence alignment and saturation mutation. Semi-rational design was proved to enhance the enzymatic properties of EC hydrolase and or urease. Directed evolution and semi-rational design could suffer heavy workload, while rational design precisely engineered certain regions or sites through structure analysis, such as charge engineering (Cui et al. 2020b), loop engineering (Yedavalli and Rao 2013) and hydrogen bond engineering (Hyun June et al. 2013). In order to address the puzzle of interactions between enzymes and organic solvents, Cui et al. revealed the general importance of the main driving force in enhanced polar variant resistance in organic solvents through molecular dynamics simulations in organic solvents, solving the problem of how to reasonably restore/improve activity and resistance through engineering (Cui et al. 2020a).

However, in addition to CompassR (Cui et al. 2021), there is a lack of general modification methods to improve the organic solvent tolerance of enzymes, especially the search for “sensitive sites” of organic solvent. High pressure could cause protein conformation changes, and different regions of protein show different responses to pressure, with different local isothermal compression coefficients (*β*_T_). *β*_T_ is the derivative of volume to pressure, which reflects the correlation between the change rate of volume with pressure and the structure fluctuation, hydrophobic action and stability of protein. Based on this, we previously developed a strategy based on isothermal compression coefficient (*β*_T_) perturbation engineering (ICPE), combined with high-pressure molecular dynamics simulation, to rationally screen "high-fluctuation" regions to obtain stable and active mutants (Zheng et al. 2022).

In this study, we innovatively combined the enzyme modification strategy based on ICPE with diphasic molecular dynamic simulation to generate dHP-MD strategy, which aimed at targeting enzyme mutants with improved activity and stability, especially organic solution resistance. dHP-MD could not only improve the stability of temperature, but also screen out the sites with poor stability in ethanol solvent. In addition, dHP-MD could also observe hydration layer change of the enzyme in organic solvent to deeply analyze the mechanism of improved organic solvent tolerance of enzyme mutants. For example, in the triple mutant H68A/K70R/S325N, the number of ethanol molecules around the residues K70R and S325N decreased (Fig. 4D), indicating that the K70 and S325 were the sensitive sites that were easily affected by ethanol. K70R and S325N mutation protected the hydrated shell with increased ethanol resistance. For H68A mutation, enzymatic activity of single mutant increased the most although the number of ethanol molecules around the H68 residue increased with negative hydrated shell (Fig. 2A, D), underlying that the dHP-MD strategy could be expected to help counteract the trade-off between organic solvent stability and activity. Although AmdA I97L/G195A reported by Kang et al. shared over 90% of tolerance in 5–20% ethanol (v/v), its enzyme activity was lower than that of the EC hydrolase in our research (Kang et al. 2021) Moreover, BpUrease L253P/L287N reported by Liu et al. (Liu et al. 2018). displayed higher affinity for urea, thus the hydrolysis ability to EC would be greatly disturbed with urea and EC existed simultaneously. In addition, dHP-MD used in this research could be more efficient than empirical case by case analysis based on static sequence alignment and structural insight for in-depth targeting mutants with improved organic solvent tolerance (Table S3), which helped form a universal and convenient engineering strategy.

Furthermore, we listed three possible criteria for mutant screening for organic solvent resistance: (i) Model the protein and simulate it under set pressure and organic solvent conditions (Yu and Dalby 2020); (ii) Analyze the simulation results, and target the unstable region by secondary structure grouping and isothermal compression coefficient calculation. (iii) Select the amino acid residues in the selected region for virtual mutation and calculate the mutation sites with reduced free energy and increased stability for experimental verification. However, there were still some limitations need to be settled. The simulated conditions in this study were relatively mild which could not meet the practical conditions in the diphasic environment. Furthermore, various strategies based on computational aids such as de novo design and/or machine learning to validate the relationship between certain structures and organic tolerance. Overall, dHP-MD was proved as an effective approach to screen out mutants of EC hydrolase with better enzyme property, such as specific activity and stability especially organic tolerance, which could provide meaningful accesses to be applied in fine engineering of other enzymes.

## Supplementary Information

**Additional file 1: Table S1.** Primer pairs used for site-directed mutagenesis of EC hydrolase by PCR. **Table S2.** Molecular dynamic constants of single-point mutations, double-point mutations and three-point mutations. **Table S3.** Comparison of mutation and properties of part of EC hydrolase and urethanase. **Figure S1.** RMSF of protein structure under 1 bar and 10% (v/v), 50% (v/v), 100% (v/v) ethanol. **Figure S2.** RMSF of protein structure under 500 bar and 10% (v/v), 50% (v/v), 100% (v/v) ethanol. **Figure S3.** RMSF of protein structure under 1000 bar and 10% (v/v), 50% (v/v), 100% (v/v) ethanol. **Figure S4.** Optimal temperature of the double mutant proteins. **Figure S5.** Ethanol tolerance of the double mutant proteins. **Figure S6.** RMSF of the triple mutant protein structure under 1 bar and 50% (v/v) ethanol. **Figure S7.** Hydrophilic accessible surface area and hydrophobic accessible surface area of WT and triple mutant enzyme.

## Data Availability

The research data generated and/or analyzed during the current study are available upon reasonable request.
