# Supplementary material for: Improving ethanol tolerance of ethyl carbamate hydrolase by diphasic high pressure molecular dynamic simulations
Source: AMB Express. 2023 Mar 15;13:32. doi: 10.1186/s13568-023-01538-7 (PMC10017909; doi:10.1186/s13568-023-01538-7)
Supplement: Supplementary file 1 — Additional file 1: Table S1. Primer pairs used for site-directed mutagenesis of EC hydrolase by PCR. Table S2. Molecular dynamic constants of single-point mutations, double-point mutations and three-point mutations. Table S3. Comparison of mutation and properties of part of EC hydrolase and urethanase. Figure S1. RMSF of protein structure under 1 bar and 10% (v/v), 50% (v/v), 100% (v/v) ethanol. Figure S2. RMSF of protein structure under 500 bar and 10% (v/v), 50% (v/v), 100% (v/v) ethanol. Figure S3. RMSF of protein structure under 1000 bar and 10% (v/v), 50% (v/v), 100% (v/v) ethanol. Figure S4. Optimal temperature of the double mutant proteins. Figure S5. Ethanol tolerance of the double mutant proteins. Figure S6. RMSF of the triple mutant protein structure under 1 bar and 50% (v/v) ethanol. Figure S7. Hydrophilic accessible surface area and hydrophobic accessible surface area of WT and triple mutant enzyme. [file 13568_2023_1538_MOESM1_ESM.docx]

**Improving ethanol tolerance of ethyl carbamate hydrolase by diphasic high pressure molecular dynamic simulations**

*Qijia Zan^a #^, Mengfei Long ^a #^, Nan Zheng ^a^, Zehua Zhang ^a^, Huimin Zhou ^a^,* *Xinjie Xu^a^，Tolbert Osire^b^ ,Xiaole Xia^a *^*

a Key Laboratory of Industrial Biotechnology, Ministry of Education, School of Biotechnology, Jiangnan University, Wuxi 214122, Jiangsu, China

b Faculty of Biology, Shenzhen MSU-BIT University, Shenzhen 518172, Guangdong,

China

***^*^*** Corresponding author

Email: xiaolexia@jiangnan.edu.cn

***^#^*** Authors contributed equally to this work

Content

[Table S1. Primer pairs used for site-directed mutagenesis of EC hydrolase by PCR 3](#_Toc128397520)

[Table S2. Molecular dynamic constants of single-point mutations, double-point mutations and three-point mutations 4](#_Toc128397521)

[Table S3. Comparison of mutation and properties of part of EC hydrolase and urethanase. 5](#_Toc128397522)

[Figure S1. RMSF of protein structure under 1 bar and 10% (v/v), 50% (v/v), 100% (v/v) ethanol. 6](#_Toc128397523)

[Figure S2. RMSF of protein structure under 500 bar and 10% (v/v), 50% (v/v), 100% (v/v) ethanol. 7](#_Toc128397524)

[Figure S3. RMSF of protein structure under 1000 bar and 10% (v/v), 50% (v/v), 100% (v/v) ethanol. 8](#_Toc128397525)

[Figure S4. Optimal temperature of the double mutant proteins. 9](#_Toc128397526)

[Figure S5. Ethanol tolerance of the double mutant proteins. 10](#_Toc128397527)

[Figure S6. RMSF of the triple mutant protein structure under 1 bar and 50% (v/v) ethanol. 11](#_Toc128397528)

[Figure S7. Hydrophilic accessible surface area and hydrophobic accessible surface area of WT and triple mutant enzyme. 12](#_Toc128397529)

## **Table S1.** Primer pairs used for site-directed mutagenesis of EC hydrolase by PCR

| Mutants | Primers |
| --- | --- |
| A321I | Forward: 5’-GAGGCAGCAATTATACACCATTCAGATTTACAAAAGAG-3' |
|  | Reverse: 5’-ATGGTGTATAATTGCTGCCTCTGAAAGAGACG-3' |
| S325F | Forward: 5’-ATACACCATTTTGATTTACAAAAGAGACCACAAG-3' |
|  | Reverse: 5’-CTTTTGTAAATCAAAATGGTGTATAGCTGCTGCCTCTG-3' |
| S325Y | Forward: 5’-ATACACCATTATGATTTACAAAAGAGACCACAAG-3' |
|  | Reverse: 5’-CTTTTGTAAATCATAATGGTGTATAGCTGCTGCCTCTG-3' |
| S325N | Forward: 5’-ATACACCATAATGATTTACAAAAGAGACCACAAG-3' |
|  | Reverse: 5’-CTTTTGTAAATCATTATGGTGTATAGCTGCTGCCTCTG-3' |
| Q332E | Forward: 5’-CAAAAGAGACCAGAAGATTTTGGTGACGATATTCG-3' |
|  | Reverse: 5’-CACCAAAATCTTCTGGTCTCTTTTGTAAATCTG-3' |
| Q332G | Forward: 5’-CAAAAGAGACCATGTGATTTTGGTGACGATATTCG-3' |
|  | Reverse: 5’-CACCAAAATCACATGGTCTCTTTTGTAAATCTG-3' |
| H68A | Forward: 5’-CCAAGGTGCATATAAGGGTATGTATCATGGGATACCTAT -3' |
|  | Reverse: 5'- CCTTATATGCACCTTGGAGAATCTCCTGTTCAAC -3' |
| H68L | Forward: 5'- CCAAGGTCTGTATAAGGGTATGTATCATGGGATACCTAT -3' |
|  | Reverse: 5'- CCTTATACAGACCTTGGAGAATCTCCTGTTCAAC -3' |
| H68M | Forward: 5'- CCAAGGTATGTATAAGGGTATGTATCATGGGATACCTAT -3' |
|  | Reverse: 5'- CCTTATACATACCTTGGAGAATCTCCTGTTCAAC -3' |
| H68K | Forward: 5'- CCAAGGTAAATATAAGGGTATGTATCATGGGATACCTAT -3' |
|  | Reverse: 5'- CCTTATATTTACCTTGGAGAATCTCCTGTTCAAC -3' |
| H68Y | Forward: 5'- CCAAGGTTATTATAAGGGTATGTATCATGGGATACCTAT -3' |
|  | Reverse: 5'- CCTTATAATAACCTTGGAGAATCTCCTGTTCAAC -3' |
| K70R | Forward: 5'- CAAGGTCATTATCGTGGTATGTATCATGGGATACCTATGGC -3' |
|  | Reverse: 5'- CCCATGATACATACCACGATAATGACC -3' |
| A178L | Forward: 5'- AGATACACTGGGCTCTATTCGAATTCCATCTTCT -3' |
|  | Reverse: 5'- ATAGAGCCCAGTGTATCTGTCCC -3' |

Note: Mutation sites are indicated by lines.

## **Table S2.** Molecular dynamic constants of single-point mutations, double-point mutations and three-point mutations

|  | *K*_m_  (mM) | *V*_max_  (μmol·min^-1^.mg^-1^) | *K*_cat_  (s^-1^) | *K*_cat_ /*K*_m_  (s^-1^·M^-1^) |
| --- | --- | --- | --- | --- |
| WT | 37.67±3.55 | 2.22±0.07 | 18509.92±4341.41 | 491.42 |
| A321I | 34.44±3.12 | 0.49±0.09 | 4056.7±969.53 | 117.77 |
| S325F | 60.12±2.97 | 1.45±0.16 | 12118.52±1897.12 | 201.57 |
| S325Y | 90.79±2.67 | 0.93±0.21 | 7827.79±850.82 | 86.22 |
| S325N | 45.52±9.41 | 1.96±0.17 | 16366.99±977.41 | 359.56 |
| Q332E | 54.35±2.37 | 2.49±1.10 | 20776.13±5046.13 | 382.26 |
| Q332G | 35.2±14.65 | 4.89±0.46 | 40791.85±417.25 | 1158.86 |
| H68A | 31.38±5.61 | 5.98±0.41 | 49874.79±2292.87 | 1589.33 |
| H68L | 46.84±2.08 | 1.75±0.92 | 14609.85±1889.55 | 311.92 |
| H68M | 56.64±1.36 | 3.15±0.53 | 26250.44±1965.52 | 463.46 |
| H68K | 66.22±1.42 | 2.78±1.22 | 23162.57±1636.48 | 349.80 |
| H68Y | 84.22±5.06 | 2.87±0.29 | 23941.35±5171.11 | 284.26 |
| K70R | 33.38±5.67 | 3.78±1.01 | 31517.63±1408.41 | 944.16 |
| A178L | 96.78±6.09 | 3.97±1.18 | 33146.88±7908.62 | 342.48 |
| H68A/K70R | 60.47±3.71 | 1.98±0.37 | 17741.94±3315.41 | 293.4 |
| H68A/A178L | 57.93±4.88 | 0.57±0.056 | 6418.92±630.63 | 110.8 |
| H68A/S325N | 33.27±2.31 | 3.77±0.97 | 26180.56±6736.11 | 786.91 |
| K70R/A178L | 56.33±4.27 | 1.92±0.76 | 20512.82±8119.66 | 364.15 |
| K70R/S325N | 40.82±3.56 | 4.33±0.54 | 17182.54±2142.86 | 420.93 |
| K70R/Q328C | 83.21±2.88 | 0.97±0.32 | 9821.79±3240.18 | 118.04 |
| A178L/S325N | 58.56±6.32 | 2.83±0.19 | 17705.2±1188.69 | 302.33 |
| A178L/Q328C | 62.78±1.73 | 1.66±0.78 | 11823.36±5555.56 | 188.32 |
| S325N/Q328C | 63.56±5.32 | 2.15±0.26 | 18470.79±2233.68 | 290.58 |
| K70R/A178L/S325N | 59.59±1.96 | 2.63±0.45 | 21959.82±2283.94 | 368.50 |
| K70R/S325N/Q328C | 84.64±2.06 | 2.28±0.26 | 19000.36±1666.277 | 224.48 |
| H68A/K70R/Q328C | 67.16±3.08 | 1.67±0.35 | 13934.93±2067.77 | 207.48 |
| H68A/K70R/S325N | 28.2±4.41 | 2.54±0.03 | 15484.74±175.08 | 549.10 |
| H68A/S325N/Q328C | 100.17±31.9 | 1.84±0.33 | 15317.27±1874.76 | 152.91 |
| H68A/K70R/A178L | 53.47±5.4 | 0.99±0.03 | 8281.45±2542.95 | 154.87 |
| H68A/A178L/Q328C | 42.7±3.50 | 1.01±0.05 | 8430.55±194.87 | 197.45 |
| A178L/S325N/Q328C | 48.82±1.95 | 1.59±0.04 | 13219.7±1559.43 | 270.76 |
| H68A/A178L/S325N | 57.2±2.71 | 2.33±0.17 | 21917.94±2457.41 | 383.20 |

## Table S3. Comparison of mutation and properties of part of EC hydrolase and urethanase.

| EC hydrolase and variants | Source | Methods for screening mutants | Enzyme activity | Ethanol tolerance of mutant | Reference literature |
| --- | --- | --- | --- | --- | --- |
| AmdA  I97L/G195A | *Agrobacterium tumefaciens* d3 | Multiple sequence alignment and structural analysis. | The wild-type enzyme activity of this protein was 0.62 U/mg, which was 3.1-fold increase for the mutant. | The wild-type ethanol tolerance of this protein was >90% in 5-20% (v/v), which was 1.5-fold increase for the mutant. | (Yao et al. 2022) |
| BpUrease  L253P/L287N | *Bacillus paralicheniformis* ATCC 9945A | Site-specific saturation mutation was carried out in the catalytic pocket and substrate inlet and outlet region | The wild-type enzyme activity of this protein was 23 U/mg, which was 10.7-fold increase for the mutant. | The enzyme activity of more than 70% can be maintained in the condition of 15% (v/v) ethanol and 37°C for 2 h | (Liu et al. 2018) |
| EC hydrolase  Q328C | *L. fusiformis* SO2 | B-factor value analysis. | Not specified | Under the condition of 5% (v/v) ethanol, the residual enzyme activity of the mutant was 40.2%, which was 13% higher than that of wild enzyme. | (Liu et al. 2016) |
| EC hydrolase  H68A/K70R/S325N | *L. fusiformis* SO2 | dHP-MD | The specific activity of triple mutant was 6.46 U/mg, which was 3.42-fold higher than that of WT | Relative enzyme activity under 20% (v/v) was 41.16%, which was 5.02-fold higher than WT. | This study |

## Figure S1. RMSF of protein structure under 1 bar and 10% (v/v), 50% (v/v), 100% (v/v) ethanol.


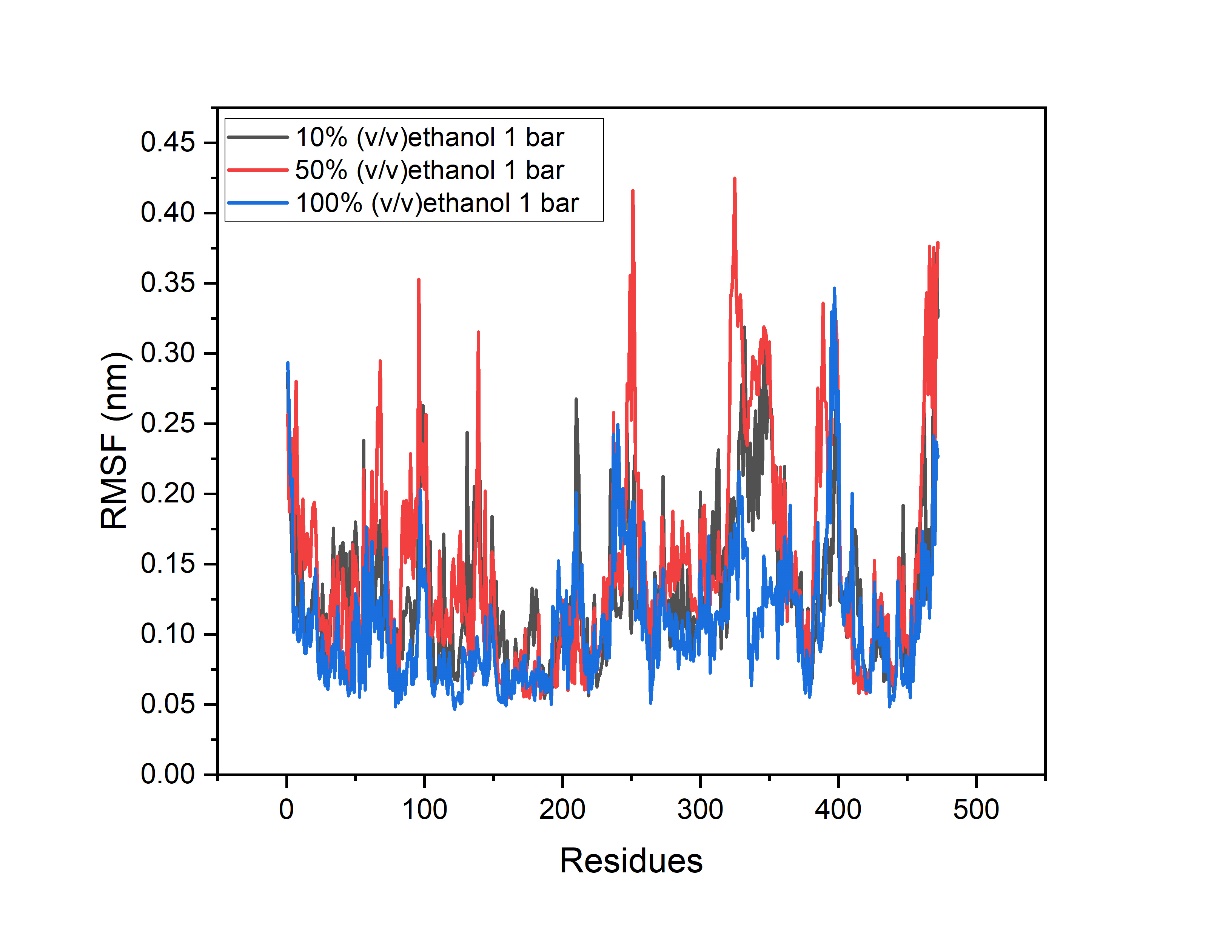


## **Figure S2.** RMSF of protein structure under 500 bar and 10% (v/v), 50% (v/v), 100% (v/v) ethanol.


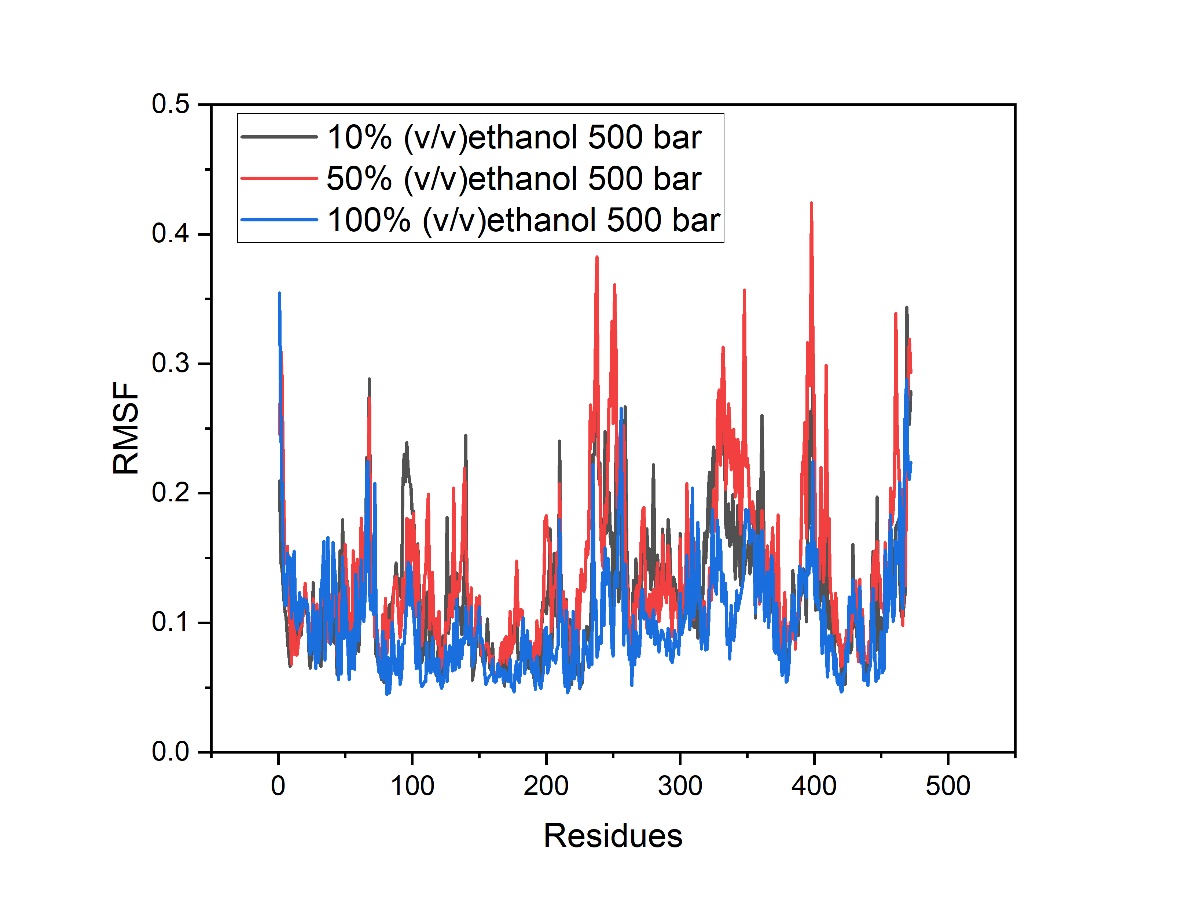


## Figure S3. RMSF of protein structure under 1000 bar and 10% (v/v), 50% (v/v), 100% (v/v) ethanol.


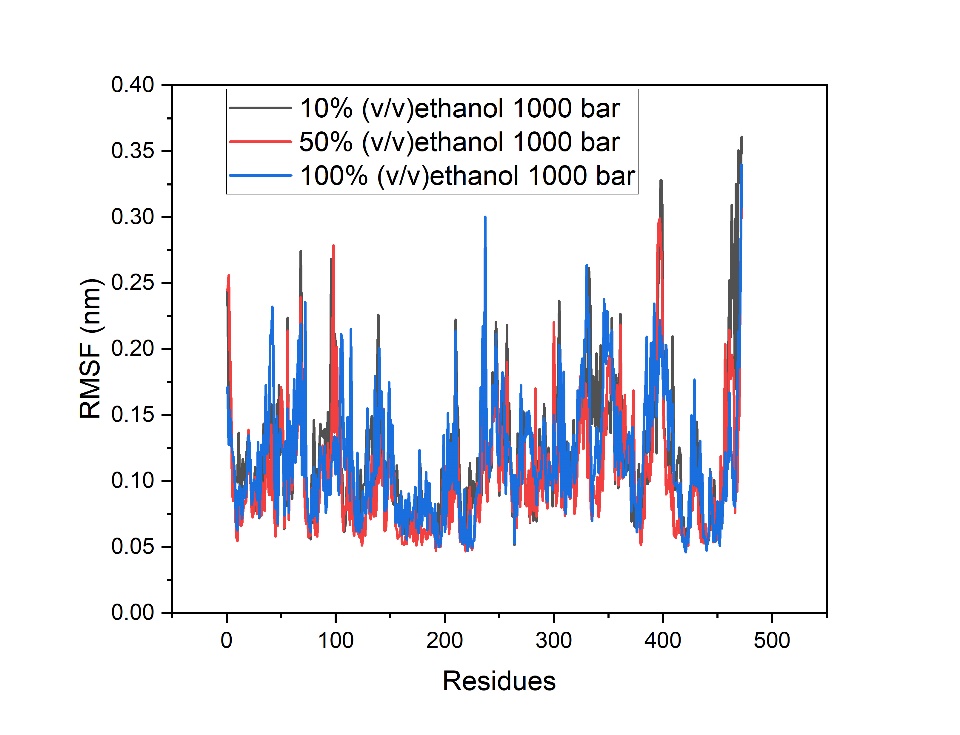


## Figure S4. Optimal temperature of the double mutant proteins.


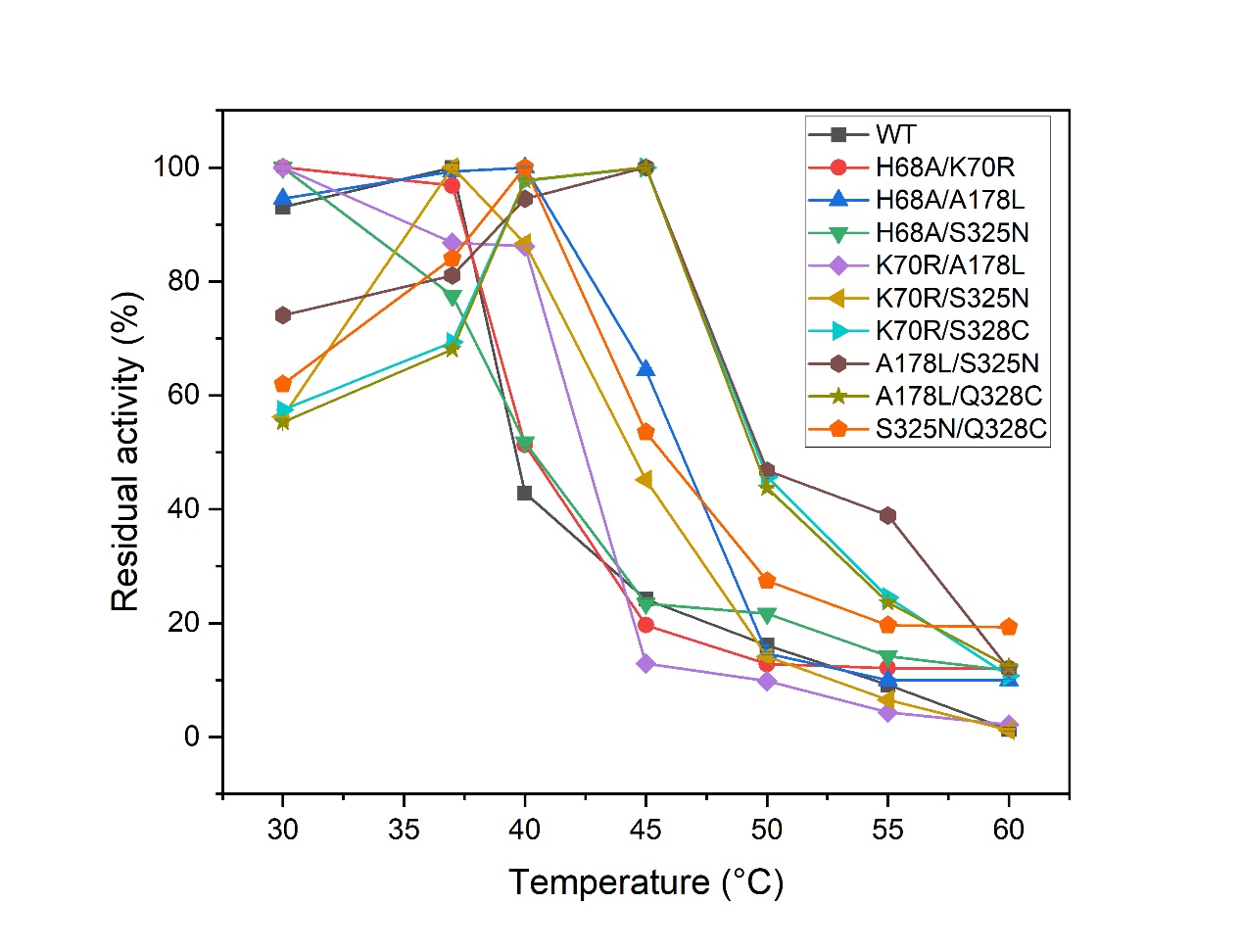


## **Figure S5.** E**thanol tolerance of the double mutant proteins.**


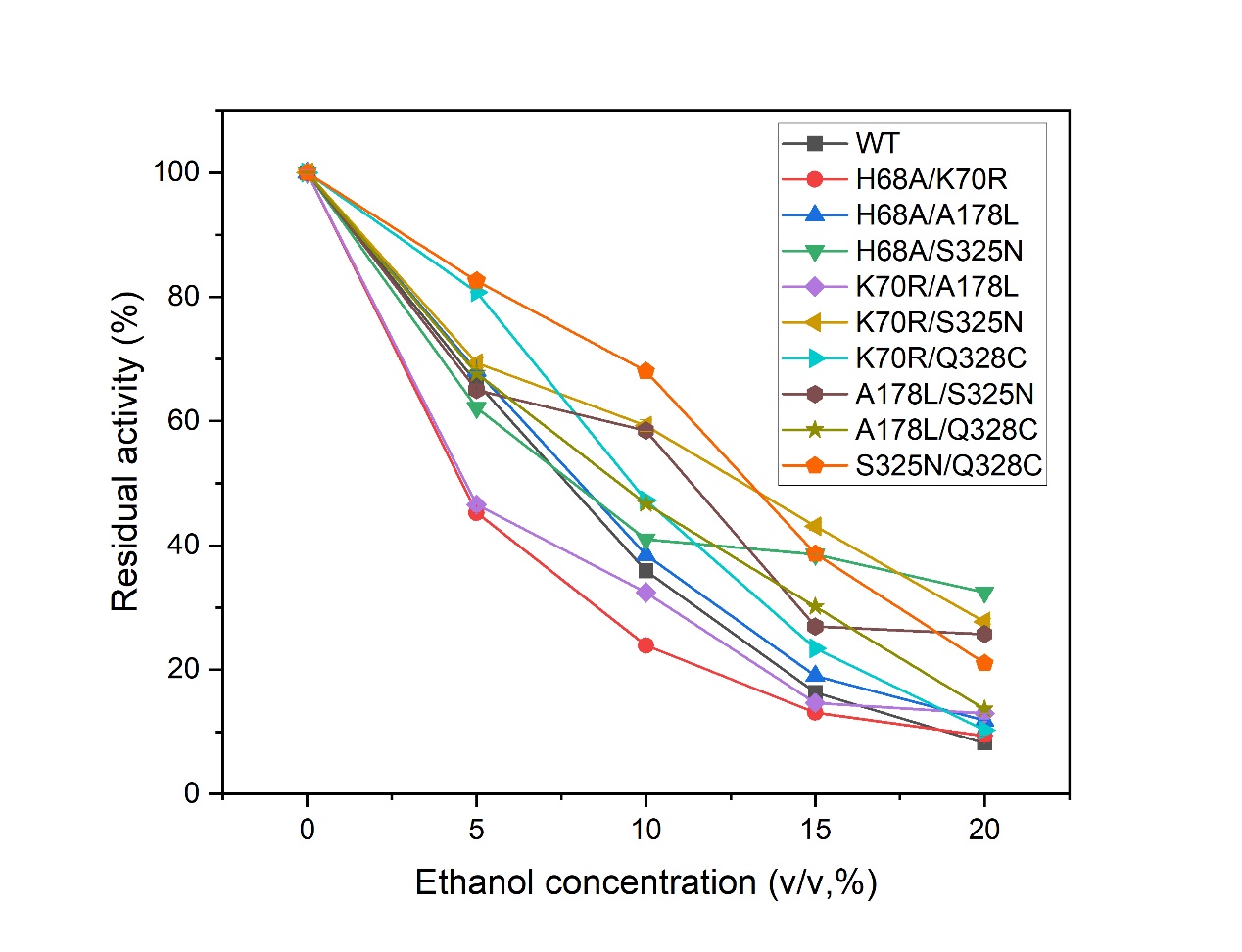


## Figure S6. RMSF of the triple mutant protein structure under 1 bar and 50% (v/v) ethanol.


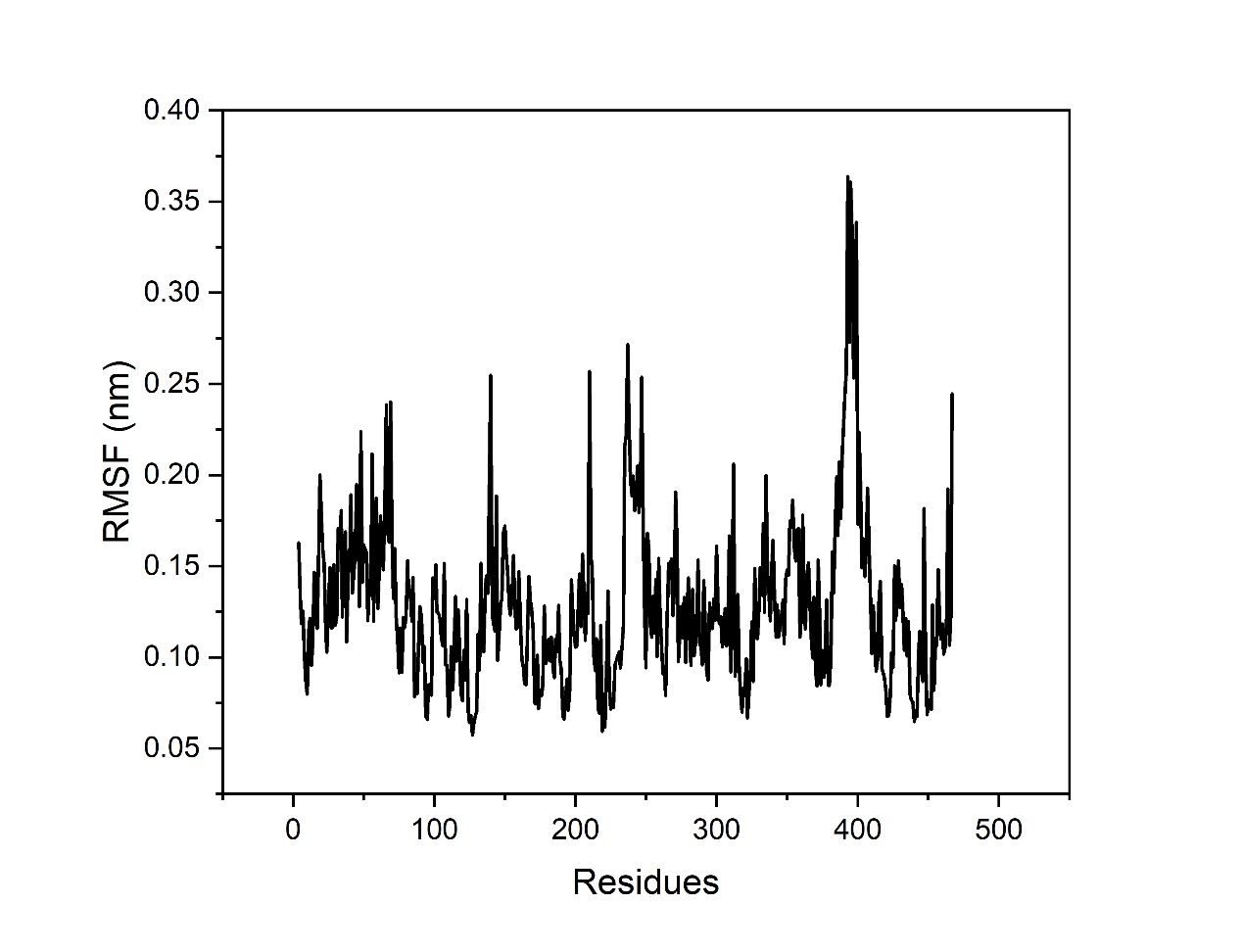


## Figure S7. Hydrophilic accessible surface area and hydrophobic accessible surface area of WT and triple mutant enzyme.


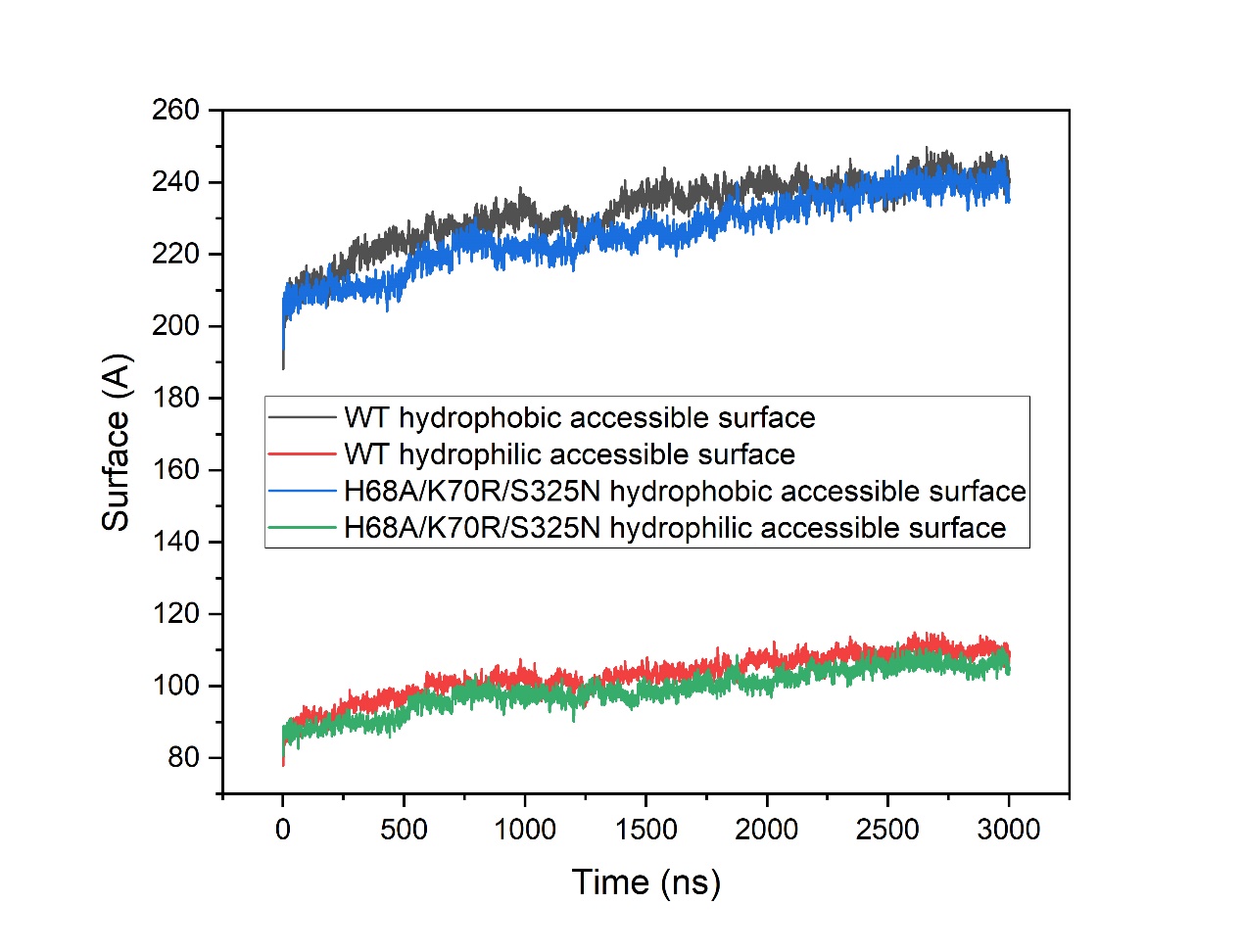


**Reference**

Liu QT, Yao XH, Liang QX, Li JH, Fang F, Du GC, Kang Z (2018) Molecular engineering of *Bacillus paralicheniformis* acid urease to degrade urea and ethyl carbamate in model chinese rice wine. J. Agric. Food Chem 66(49):13011-13019. doi:10.1021/acs.jafc.8b04566.

Liu XH, Fang F, Xia XL, Du GC, Chen J (2016) Stability enhancement of urethanase from *Lysinibacillus fusiformis* by site-directed mutagenesis. Chin. J. Agric. Biotechnol. 32(9):1233-1242. doi:10.13345/j.cjb.150527.

Yao XM, Kang TT, Pu ZJ, Zhang T, Lin JP, Yang LR, Yu HR, Wu MB (2022) Sequence and structure-guided engineering of urethanase from *Agrobacterium tumefaciens* d3 for improved catalytic activity. J. Agric. Food Chem 70(23):7267-7278. doi:10.1021/acs.jafc.2c01406.
